# Osteoblasts-derived exosomes as potential novel communicators in particle-induced periprosthetic osteolysis

**DOI:** 10.1016/j.mtbio.2024.101189

**Published:** 2024-08-06

**Authors:** Wanderson de Souza, S. Gemini-Piperni, Carolina Ruivo, Nuno Bastos, Sofia Almeida, Daniel Lopes, Patricia Cardoso, Maria Jose Oliveira, D. Rick Sumner, Ryan D. Ross, Joshua J. Jacobs, Jose Mauro Granjeiro, Maria Helena Fernandes, Luis A. Rocha, Sonia Melo, Ana R. Ribeiro

**Affiliations:** a Directory of Life Sciences Applied Metrology, National Institute of Metrology Quality and Technology, Rio de Janeiro, Brazil; b Postgraduate Program in Biotechnology, National Institute of Metrology Quality and Technology, Rio de Janeiro, Brazil; c Postgraduate Program in Translational Biomedicine, University Grande Rio, Duque de Caxias, Brazil; d Labεn Group, Federal University of Rio de Janeiro (UFRJ), Rio de Janeiro, Brazil; e I3S-Institute for Research and Innovation in Health, University of Porto, Portugal, Porto, Portugal; f Department of Orthopedic Surgery of RUSH University, Chicago, USA; g Department of Anatomy & Cell Biology of RUSH University, Chicago, USA; h Dental School, Fluminense Federal University, Niterói, Brazil; i Faculty of Dental Medicine, University of Porto, Porto, Portugal; j LAQV/REQUIMTE, University of Porto, Porto, Portugal; k proMetheus, Escola Superior de Tecnologia e Gestão, Instituto Politécnico de Viana do Castelo, Viana do Castelo, Portugal; l IBTN/EURO – European Branch of the Institute of Biomaterials, Tribocorrosion and Nanomedicine, Izmir Institute of Technology, Izmir, Turkey; m Nanosafety group, International Iberian Nanotechnology Laboratory (INL), Braga, Portugal

**Keywords:** Titanium dioxide, Nanoparticles, Exosomes, Osteoblasts, Macrophages, Inflammation, Osteolytic patients

## Abstract

The inflammatory response to wear particles derived from hip prothesis is considered a hallmark of periprosthetic osteolysis, which can ultimately lead to the need for revision surgery. Exosomes (Exos) have been associated with various bone pathologies, and there is increasing recognition in the literature that they actively transport molecules throughout the body. The role of wear particles in osteoblast-derived Exos is unknown, and the potential contribution of Exos to osteoimmune communication and periprosthetic osteolysis niche is still in its infancy. Given this, we investigate how titanium dioxide nanoparticles (TiO_2_ NPs), similar in size and composition to prosthetic wear particles, affect Exos biogenesis. Two osteoblastic cell models commonly used to study the response of osteoblasts to wear particles were selected as a proof of concept. The contribution of Exos to periprosthetic osteolysis was assessed by functional assays in which primary human macrophages were stimulated with bone-derived Exos. We demonstrated that TiO_2_ NPs enter multivesicular bodies, the nascent of Exos, altering osteoblast-derived Exos secretion and molecular cargo. No significant differences were observed in Exos morphology and size. However, functional assays reveal that Exos cargo enriched in uPA stimulates macrophages to a mixed M1 and M2 phenotype, inducing the release of pro- and anti-inflammatory signals characteristic of periprosthetic osteolysis. In addition, we demonstrated the expression of uPA in exosomes derived from the urine of patients with osteolysis. These results suggest that uPA can be a potential biomarker of osteolysis. In the future, uPa may serve as a possible non-invasive biomarker to identify patients at risk for peri-implant osteolysis.

## Graphical abstract

**Figure:**
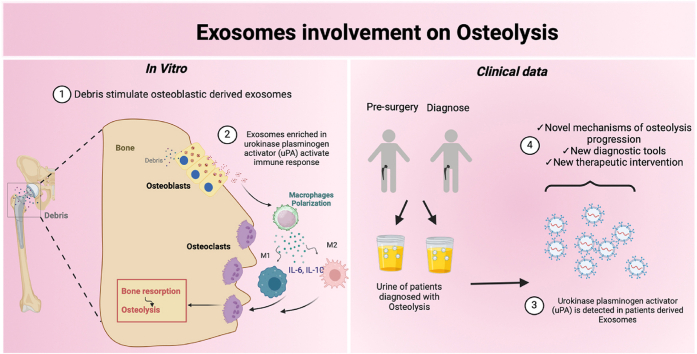

## 1 Introduction

Titanium (Ti) and its alloys are the most used biomaterials for joint replacement due to their excellent biocompatibility [1,2]. However, one of the main Ti disadvantages is its susceptibility to mechanical wear (due to cyclic loading) and corrosion (due to contact with biological fluids), which leads to the release of wear debris and corrosion products that cause local and systemic complications in patients [[3], [4], [5]]. Histopathological studies show that Ti wear particles with different sizes and crystal structures accumulate in the synovial peri-implant membrane, bone marrow, and peri-implant regions affected by fibrotic and inflammatory changes [3,4,6]. Due to its high persistence (low solubility), Ti wear particles induce a complex immune response leading to periprosthetic osteolysis (degeneration of bone causing aseptic loosening) and implant failure, requiring a revision surgery [[3], [4], [5],[7], [8], [9], [10]] that place a high financial burden on health care systems [10,11].

The main biological mechanisms underlying the response to Ti wear particles are innate immune responses, in which phagocytosis of wear particles triggers macrophage activation [12]. The reactivity of macrophages leads to a proinflammatory milieu (upregulation of proinflammatory cytokines, mainly IL-1β, IL- 6, and TNF-α) in the vicinity of bone, which disrupts its homeostasis (decreased osteoblastogenesis and increased osteoclastogenesis), resulting in periprosthetic osteolysis [5,[7], [8], [9], [10],^12^]. Although the chronic inflammatory response is mainly driven by macrophages, osteolysis is also indirectly caused by the contribution of other cell types such as osteoblasts, dendritic cells, osteoclasts, and synovial fibroblasts [3,4]. Despite osteoblasts being the major cellular players in bone homeostasis their response to wear particles is limited but critical to understand osteolysis as a whole. Furthermore, chronic inflammation can be maintained by a continuous release and diffusion of soluble mediators that drive progressive bone resorption but can also cause severe and extensive pathologies (e.g., metallosis or necrotic-appearing soft tissue masses) [13]. A warning sign is the ability of some nanoscale wear particles to cross epithelial barriers, migrate through the lymphatic or circulatory systems, and accumulate far from the site of the implant, leading to systemic toxicity [[3], [4], [5]]. Their circulation, protected from phagocytosis by patrolling monocytes, can be supported by extracellular vesicles (e.g., exosomes (Exos), which are central players in intracellular communication [14,15]. Extracellular vesicles are found in all body fluids. They carry a variable spectrum of molecules characteristic of the cells from which they originate and can alter the function and physiology of recipient cells [15,16]. Exos regulate many pathophysiological processes, including immune and inflammatory responses, and carry their enriched content (miRNAs, DNA, peptides), which promotes cell-cell communication and thus plays a vital role in mediating tissue repair and regeneration [14]. We hypothesize that in addition to the intracellular response of osteoblasts to the uptake of wear particles, the communication of cells in the periprosthetic niche may be altered, with Exos playing an essential and active role. We have previously shown that titanium dioxide nanoparticles (TiO_2_ NPs) induced the secretion of osteoblast-derived Exos that impaired osteogenic differentiation of mesenchymal stem cells [16]. However, the effects of wear debris on bone-immune cell communication are not yet known, and the detailed mechanisms underlying the biological contribution of Exos to osteolysis remain unknown. Therefore, in this work, we investigate the effect of TiO_2_ NPs on osteoblasts-derived Exos biogenesis and their involvement in inflammatory responses, which are considered hallmarks of periprosthetic osteolysis.

Exos were the focus of this study because it is well known that they provide a way for cells to get rid of unneeded or unwanted material (such as cellular contents and wear particles). They promote innate and adaptive immunity, and there is already some evidence that Exos act as mediators of chemical toxicity [[17], [18], [19]]. We have shown for the first time that osteoblast-derived Exos (derived from osteoblasts exposed to TiO_2_ NPs) stimulate human macrophages to a mixed phenotype, leading to the secretion of inflammatory cytokines that contribute to periprosthetic inflammation and subsequent osteolysis. Futhermore, we showed that osteoblasts-derived Exos were enriched in uPA and that uPA was also expressed in Exos from patients with osteolysis. Future studies revealing the precise composition of the Exos cargo (e.g., microRNA) may reveal the mechanisms behind the systemic diffusion of toxic signals through orthopedic wear debris.

## 2 Material and methods

### 2.1 Characterization of TiO_2_ NPs (mimicking nano wear debris)

Based on previous experience of the group [16,[20], [21], [22]], TiO_2_ NPs (Product No. 1317-70-0, particle size <25 nm; anatase crystal structure; surface area: 45–55 m^2^/g) (Sigma-Aldrich) were selected for this study. The characterization and dispersion of TiO_2_ NPs have been previously published [16,[20], [21], [22]]. Resuming, a stock suspension of TiO_2_ NPs was prepared in ultrapure water (concentration 2 mg/mL, pH 4). The samples were dispersed in an ultrasound (ultrasound, Q-Sonica) equipped with a 19 mm Ti tip. The sonication was performed at 32 W of power for 15 min (min) in pulse mode (8 s (sec) ON and 2 s OFF). Particle size distribution analysis was performed by dynamic light scattering (DLS) using a ZetaSizer Nano ZS (Malvern Instruments) after 24 h (h) of stabilization. The characterization of TiO_2_ NPs in the cell culture medium was performed by diluting the suspension of 2 mg/mL of NPs in Minimum Essential Medium (α-MEM, Gibco) supplemented with 10 % fetal bovine serum (V/V) (FBS, Gibco), pH 7.4. All the suspensions were also characterized by high-resolution transmission electron microscopy (HRTEM, JEOL 2100 F operating at 200 kV equipped with an X-ray Detector (EDX – Energy-dispersive X-ray spectroscopy).

### 2.2 MG63, SAOS-2 and macrophage culture

The minimal essential medium (α-MEM, Gibco) supplemented with 10 % fetal bovine serum (V/V) (FBS, Gibco) was used for the culture of osteoblasts: MG63 cells (immature osteoblasts, human) and SAOS-2 cells (mature osteoblasts, human). Osteoblast cell lines were supplied by the Rio de Janeiro Cell Bank (BCRJ), where they were packed in freezer vials and kept in liquid nitrogen. After thawing, the cells were expanded into 25 and/or 75 cm^2^ cell culture flasks (Corning). The cells used in the experiments were between 2° and 3° passages and were kept in a humidified incubator (5 % CO_2_, 37 °C). Contamination of cells with bacteria, fungi or mycoplasma was analyzed. Based on previous data of the group [16,[20], [21], [22]]. Human monocytes were isolated from buffy coats from healthy blood donors, obtained at University Hospital Center São João (CHUSJ). All studies using these human samples were approved by the CHUSJ Ethics Committee for Health (References 259 and 260/11), in agreement with the Helsinki declaration. Informed consent was obtained from all subjects. As previously described, human monocytes were isolated from buffy coats from healthy blood donors [23]. Briefly, buffy coats were centrifuged for 30 min, at 1200 g, without brake. The whitish layer containing peripheral blood mononuclear cells was collected and incubated with the RosetteSep Human Monocyte Enrichment Cocktail (StemCell Technologies), for 20 min and under rotation, following the manufacturer's instructions. This mixture was diluted (1:1) in phosphate-buffered saline (PBS 0.01 M) supplemented with 2 % FBS (Biowest), added over Histopaque-1077 (Sigma-Aldrich), and centrifuged as previously described. The intermediate layer, enriched in monocytes, was collected, and washed three times in PBS 0.01 M and centrifuged at 1300 rpm. for 6 min. On average, 90 % of isolated monocytes were CD14^+^ positive. For monocyte-macrophage differentiation, 1 x 10^6^ cell/cm^2^ monocytes were seeded on circular glass coverslips with 30 mm diameter (6-well plates, Marienfeld), in complete RPMI medium (10 % FBS and 1 % Penicillin/Streptomycin (Gibco)), supplemented with 50 ng/mL rhM-CSF (Immunotools), for 7 days. Then, the medium was renewed without rhM-CSF supplementation for 3 days. On day 10, macrophages were treated with TiO_2_ NPs (100 μg/mL) and incubated at 5 % CO_2_ at 37 °C for 72 h.

### 2.3 MG63, SAOS-2 and macrophage cell viability upon TiO_2_ NPs exposure

MG63, SAOS-2, and human macrophage cells were exposed to TiO_2_ NPs (100 μg/mL) for 72 h. Viability (MG63 and SAOS-2 density: 3 x 10^4^ cell/cm^2^/human macrophage density: 5 x 10^5^ cell/mL) was measured using the Annexin V Dead Cell Apoptosis Kit (Life and Dead Kit, Life Technologies) (through the combination of annexin V and propidium iodide (PI), it is possible to distinguish the % viable cells (Annexin V ‾/PI ‾), apoptotic cells (Annexin V⁺/PI ‾ and PI⁺) and, necrotic cells (Annexin ‾/PI⁺)) in a flow cytometer (FACSAria III, BD Biosciences), following the protocol already published [16,[20], [21], [22]]. The analysis was repeated in three independent experiments for the 3 cell types. The analysis was performed with macrophages from at least 4 different donors.

### 2.4 MG63, SAOS-2 and macrophage cell internalization upon TiO_2_ NPs exposure

The analysis of TiO_2_ NPs internalization in MG63, SAOS-2 and human macrophage cells was performed by transmission electron microscopy analysis. All samples were fixed with modified Karnovsky for 2 h at room temperature and washed with 0.1 M cacodylate buffer with 1 % uranyl acetate (diluted in water) (uranyl acetate, Sigma-Aldrich) overnight. The samples were dehydrated in a series of ethanol (Ethanol, VETEC) (30–100 %) and finally included in Epon 812 resin (EMS). Ultrathin sections (70 nm) were cut in ultramicrotome and examined under transmission electron microscopy (TEM) (Tecnai Spirit G2, FEI), and a three-dimensional reconstruction was performed using the focused ion beam (FIB) technique. At least ten cells from each group (control and 100 μg/mL) were analyzed.

### 2.5 Reactive oxygen species quantification and lysosome staining upon TiO_2_ NPs exposure

Reactive oxygen species (ROS) were identified using the 2,7-dichlorofluorescein diacetate probe (H2D-CFDA). Cells were incubated (in samples interacted with TiO_2_ NPs) with 2,7-dichlorofluorescein at an ambient concentration of 10 μM in the dark (10 min) using protocols provided by the manufacturer (Molecular Probes). Cultures were photographed on an inverted phase contrast microscope (Nikon TMS). They were quantified by measuring the fluorescence intensity measured at 488 nm and 530 nm wavelengths in a microplate reader (Synergy HT, BioTek). Lysosomes (in samples interacted with TiO_2_ NPs) were stained with 500 nM lysotracker (Life) (green) in culture medium for 15 min at 37 °C (following kit recommendations). They were incubated with DAPI (Sigma-Aldrich) for core labeling. Cultures were photographed on an inverted phase contrast microscope (Nikon TMS).

### 2.6 Isolation, purification, and characterization SAOS-2 and MG63 derived exos

#### 2.6.1 Isolation of osteoblasts derived Exos

To isolate Exos, 5 x 10^5^ cells/cm^2^ (MG63 and SAOS-2) were cultured in culture medium supplemented with 10 % FBS (Exos-free) for 72 h [24,25]. Cells were washed 3 times with PBS 0.01 M and the medium was collected and centrifuged at 4000×*g*, 4 for 10 min (Sigma). The supernatant was collected and filtered with 0.2 μm filters (Corning) and transferred to pollinator (Beckman) ultracentrifugation tubes (Beckman Optima L80-XP) using the SW41 rotor (Beckman) for 16 h at 100,000 g at 4 °C (Beckman Optima L80-XP) and the SW41 rotor (Beckman) for 6 h at 100,000 g. Exos were then washed, the pellet was resuspended in 1 mL PBS 0.01 M and another ultracentrifugation was performed (Beckman Optima L80-XP). The supernatant was obliterated, and the Exos pellet was resuspended in 100 μL of PBS 0.01 M. The same procedure (with the same number of cells) was performed after the exposure of MG63 and SAOS-2 cells to TiO_2_ NPs (5, 10, 25, 50, and 100 μg/mL) for 72 h. To remove the high number of NPs in the supernatants, the cells were washed 3 times with PBS 0.01 M and a fresh culture medium was added with 10 % FBS (Exos-free). After 24 h, the medium was collected and centrifuged as previously described. As controls, cell supernatants without interaction with NPs were used.

#### 2.6.2 Exos purification

To eliminate any trace of TiO_2_ NPs and to purify the population of Exos, a sucrose gradient was performed in the Exos suspension of both cell models. Purification of Exos was performed with a continuous sucrose gradient following the protocol already described [24,25]. The Exos were resuspended in 2 mL of 2.5 M sucrose, 20 mM HEPES/NaOH, pH 7.2. A linear gradient of sucrose (0.25M–2.0M sucrose, 20 mM HEPES/NaOH) was layered on top of the Exos suspension in an SW41 tube (Beckman). Gradients were centrifuged for 16 h at 210,000×*g* and 1 mL fractions were collected from the top of the tube. The densities were evaluated using a refractometer. Exos have been found to float at densities ranging from 1.15 to 1.19 g/mL on continuous sucrose gradients [24,25].

#### 2.6.3 The efficacy of Exos purification

The presence of Ti in purified (by sucrose gradient) Exos and non-purified Exos suspension was investigated. As a positive control, a stock solution of TiO_2_ NPs (2 mg/mL) was used, and as a negative control, PBS 0.01 M (without contact with NPs) was analyzed. Titanium (48Ti) analysis was performed using an iCAP™ Q instrument (Thermo Fisher Scientific, Bremen, Germany), equipped with a Meinhard® TQ + high-sensitivity nebulizer, a disconcerted (Peltier-cooled) cyclonic spray chamber, a Standard quartz torch and a two-cone design (nickel sample and skimmer cones). High purity argon (99.9997 %) (Gasin, Portugal) was used as a nebulizer and as a source of plasma gas. The operating parameters of the ICP-MS instrument were as follows: RF power (1550 W); plasma gas flow (14 L/min); auxiliary gas flow (0.8 L/min); and nebulizer flow rate (1.01 L/min). Elemental isotope scandium (45Sc) was used as the internal standard. Samples were diluted 1:20, and their concentrations were derived from linear regression equations representing the relationship between the analytical signal (ICPS) and the elemental concentration of the corresponding standard solution.

#### 2.6.4 Quantification, average size, and morphology of Exos

Nanoparticle Tracking Analysis (NTA) (NanoSight NS300 - Malvern Panalytical) was used to measure the size distribution and concentration of intact Exos isolated from cultures of MG63 and SAOS-2 cells. The isolated Exos were resuspended in PBS 0.01 M (diluted 3:1000) and analyzed. The protocol applied was previously described [16,24,25]. The morphology and size of Exos isolated and purified by sucrose gradient following the procedures described before were characterized by TEM [24]. To remove aggregates of proteins and improve the morphological analysis of Exos, a wash (PBS 0.01 M) was performed. Basically, after isolation and purification, Exos were resuspended in PBS 0.01 M and transferred to polyallomer tubes (Beckman) and placed in the ultracentrifuge (Beckman Optima L80-XP) using the SW41 rotor (Beckman), and centrifuged for 16 h at 100,000 g at 4 °C. After that, Exos were resuspended in 50 μL of PBS 0.01 M, and a drop of the suspension was placed onto a holey coated copper grid. Samples were contrasted in 1 % uranyl acetate and visualized in the transmission electron microscope (TEM, Tecnai Spirit G2, FEI).

#### 2.6.5 Identification of Exos surface marker

For identification of the CD63 and syntenin-1, Exos protein, 20 particles/uL solution (from MG63 and SAOS-2) without interaction with NPs were added to 10 μL of 1 x buffer (0.0625 M Tris-HCL, 2.5 % SDS, 5 % Glycerol) and frozen to −20 °C. Samples were boiled for 10 min at 95 °C and run on 12,5 % bis-polyacrylamide gel, 120 V were used to separate the proteins by molecular weight. Therefore, the gel was stained using Ponceau S staining (Sigma Aldrich, Cat# P17170). The bis-polyacrylamide gel was transferred to a nitrocellulose membrane (Millipore) at 100 V for 90 min. Membranes were blocked in PBS/0.1 % Tween-20 (PBS/T) with 5 % skim milk, incubated with CD63 (1:500, BD Pharmingen, Cat # 556019) and syntenin-1 primary antibody (1:500, Abcam, Cat# ab133267) diluted in PBS/T, and incubated overnight. The membranes were washed three times with PBS/T and incubated with blocking solution for 1 h. After this time, the membranes were incubated with conjugated secondary antibody (1:2000, R&D Systems, Cat# HAF007) and anti rabbit-horseradish peroxidase (HRP) (1:5000, Cell Signaling Technology, Cat# 7074S) followed by washing in PBS/T. Blots were developed using the ECL Plus (GE Healthcare) Western Blotting Detection System following the manufacturer's instructions.

#### 2.6.6 Identification of osteoblastic-derived Exos proteomic cargo

The proteins in Exos isolated from human osteoblasts (MG63 and SAOS-2) were analyzed. To this end, we investigated the proteins present in Exos samples obtained from the control without contact with TiO_2_ NPs of both cell models and in samples obtained after interaction with TiO_2_ NPs for 72 h. Samples were purified by sucrose gradient and washed to remove contaminating proteins. The concentration of Exos was normalized for all points (about 90 μg/mL protein at each point analyzed). An enzymatic digestion was then performed using 0.2 μg trypsin (Promega) diluted in ammonium bicarbonate (50 Mm, 30 min in overnight ice at 37 °C). The extracted peptide mixture was lyophilized in 1 % formic acid and transferred to StageTip (C18). A drying process was applied. Then 25 μL of methane acid (1 %) (Sigma) was added. Samples were analyzed on a mass spectrometer (MS) (EDT-enabled Orbitrap Velos) (Thermo-Fisher Scientific) coupled to an EASY-nLC (Proxeon Biosystem) system using a Proxeon nanoelectrosplay source. The peptides were separated on a gradient of 2–90 % acetonitrile in 1 % methane acid in a PicoFrit analytical column (20 cm x ID75 μm, 5 μm particle size), with a flow of 300 nL/min for 27 min. The nanoelectrosplay voltage and temperature were adjusted to 2.2 kV and 275 °C, respectively. The method configured for LTQ Orbitrap Velos was data-dependent analysis (ADD). SM scanning spectra (*m*/*z* 300–1600) were acquired on the Orbitrap analyzer after accumulation to a target value of 1 and 6, and the Orbitrap resolution was adjusted to r = 60,000. Thus, the 20 most intense peptide ions with charge states ≥2 were sequentially isolated to a target value of 5000 and fragmented into the linear low-energy CID ion trap (35 % normalized collision energy). The signal threshold for triggering an SM/SM event has been set to 1000 counts. Dynamic deletion was enabled with a size list of 500, and the deletion duration was 60 s. The activation Q value was 0.25, and the activation time was 10 ms. Data were obtained using the Xcalibur software package, and the samples were analyzed in three biological replicates. Peak lists (msf) were generated from files containing raw data using Proteome Discoverer version 1.3 (Thermo-Fisher scientific) with the Sequest search engine and searched against taxon H sapiens from the UniProtKB/SwissProt database (release 2016_04) with carbamidomethylation as a fixed modification. The Software (version Scaffold_4.5.1, Proteome Software Inc., Portland, OR) was used to validate SM/SM-based peptide and protein identifications. Peptide identifications have been accepted when a probability greater than 99.0 % can be established to achieve an FDR of less than 1.0 % by the Scaffold local FDR algorithm. Protein identifications were accepted when a probability greater than 80.0 % could be established to achieve an FDR less than 1.0 % and contained at least one identified peptide. Protein probabilities were assigned by the Protein Prophet algorithm. Proteins containing similar peptides that could not be differentiated based on the SM/SM analysis alone were pooled. Proteins were noted with GO terms of H sapiens filtered (gene_association.goa_cow.gz, downloaded 06-Apr-2016). We have presented the results of this analysis in a previous publication [16]. For new approaches, we used ShinyGO (version 0.77) and FunRich (version 3.1.4) software to update the gene ontology classification based on their involvement in the KEGG Pathway and classified biological processes through the Uniprot database for taxonomic *Homo sapiens*. Considering the control versus treatment (p 0.01), we obtained the proteins that increased and decreased after the treatment of TiO_2_ NPs in the studied cells. Results are the mean ± standard deviation of triplicate independent experiments.

### 2.7 Functional tests: effect of osteoblasts-derived Exos on macrophages

Macrophages were treated with different concentrations of Exos obtained from MG63 cells at the following concentrations: Exos obtained without stimulation with TiO_2_ NPs (named as ExosMG63_1 (4 x 10^3^ particles/mL), ExosMG63_2 (4 x 10^5^ particles/mL), and ExosMG63_3 (4 x 10^7^ particles/mL), the same concentrations were used for Exos obtained after stimulation with TiO_2_ NPs (named as ExosMG63 + NPs_1:3). Macrophages were treated with RPMI medium supplemented with 10 % FBS free Exos and 1 % penicillin/streptomycin and incubated at 5 % CO_2_ and 37 °C for an additional 72 h. As a control, we examined macrophages exposed to neither Exos nor NPs (control) and exposed to 100 μg/mL TiO_2_ NPs for 72 h (NPs 100 μg/mL).

#### 2.7.1 Macrophage morphology and cytoskeleton evaluation

Upon Exos’ treatments and TiO_2_ NPs (as a control), macrophages were fixed in 4 % paraformaldehyde, quenched with 50 mM NH_4_Cl for 10 min and, after PBS 0.01 M washes, permeabilized during 5 min with 0.1 % Triton X-100. Blocking was performed for 30 min with 5 % bovine serum albumin (Sigma–Aldrich). Coverslips were incubated for 1 h with monoclonal anti-a-tubulin (Sigma–Aldrich) antibody, washed, and incubated, for an additional 45 min with goat anti-mouse AlexaFluor-488-conjugated-secondary antibody (ThermoFisher Scientific). F-actin was stained with Alexa Fluor 569 Phalloidin (ThermoFisher Scientific) for 20 min. Coverslips were washed and mounted with Vectashield + DAPI (VectorLaboratories). Cell morphology was analyzed under a Leica TCS-SP5 AOBS spectral confocal microscope (Wetzlar, Germany).

#### 2.7.2 Macrophage cytokine profile activation

The human macrophage culture supernatant media was collected following treatment with MG63 Exos and TiO_2_ NPs (control). Media were collected and centrifuged at 1200 rpm for 5 min to remove cell debris. The concentration of IL-6, IL-10, IL-1b, TNF-a, IFN-g and TGF-b was determined by ELISA (Biolegend), according to the manufacturer's instructions. As a control, media without the addition of NPs were also evaluated.

#### 2.7.3 Macrophage surface markers

For the flow cytometry analysis of cell surface receptor expression, upon Exos treatments and TiO_2_ NPs (additional control), macrophages were detached by incubation with Accutase (Grisp) at 37 °C for 30 min and harvested by gentle scraping. The cells were washed and resuspended in FACS buffer (PBS 0.01 M, 2 % FBS, 0.01 % sodium azide) and stained with specific conjugated fluorophore-antibodies, in the dark, for 45 min at 4 °C. Macrophages were incubated with the following antibodies: anti-human CD14-APC (clone MEM-18; Immunotools), CD163-PE (clone GHI/61; R&D Systems) and CCR7-PerCPCy5.5 (clone G043H7; Biolegend). Isotype-matched antibodies were used as negative controls. Cells were acquired on a FACSCanto flow cytometer (BD Biosciences) and analyzed with FlowJo software (v10.6.1). The median fluorescence intensity (MFI) was calculated by subtracting the respective isotype control intensity.

### 2.8 Identification of uPA in patient samples

The Institutional Review Board for Human Research approved the study design. Every patient completed a consent form granting authorization for biofluids to be stored for use in research projects in the future. All studies using these human samples were approved by the Rush Medical Center Ethics Committee (references 14102805) following the Declaration of Helsinki. Informed consent was obtained from all donors. This study included a total of 6 patients who were receiving primary total hip replacement by surgeons at Midwest Orthopaedics at Rush. Every patient got a titanium alloy stem. Six of the patients who were included were assigned to the osteolysis group after radiographic evidence of peri-implant osteolysis appeared, either next to the hip stem or the acetabular component (Demographic data in Figure additional files 1). Patients had routine radiographic evaluations in the region and urine samples were synchronized according to the first reported radiographic lesion. Urine samples from 6 patients diagnosed with particle-induced osteolysis were collected at different time points: three months before hip replacement surgery (pre-surgery), and upon diagnosis of osteolysis (radiographic evidence). Upon material transfer agreement, samples were sent to Portugal and the isolation, quantification and average size of Exos were performed as previously described (see 2.6). Characterization of syntenin-1 was performed as described in section 2.6.5. The following procedure was used to characterize Alix and uPA: Proteins were extracted from extracellular vesicles using a SDS2.5 %/8M urea (Sigma-Aldrich) lysis buffer, supplemented with complete (Roche) and phenylmethylsulphonyl fluoride (PMSF, Sigma) and then incubated for 30 min on ice. Then, the proteins were incubated with laemmli buffer without β-mercaptoethanol (ratio 4:1) for 10 min at 95 °C. Proteins were separated on a 12,5 % SDS-PAGE (sodium dodecylsulphate-polyacrylamide gel electrophoresis) gel and transferred to 0.2 μm nitrocellulose membranes (Millipore) using a wet electrophoretic transfer (GE Healthcare) at 100V for 90 min 4 °C. Subsequently, the membranes were blocked in 5 % non-fat dry milk in PBS (phosphate-buffered saline) 1X/0,1 % Tween 20 (Sigma-Aldrich) for 1 h at room temperature with agitation. Membranes were then incubated overnight at 4 °C with primary antibodies diluted in the same solution as used for blocking. The following primary antibodies were used: Alix (1:500, Cell Signaling Technology Cat# 2171) for extracellular vesicles detection; and uPA (1:500, Cat# MAB807). After washing three times, for 10 min each, with PBS-T 0,1 %, membranes were incubated for 1 h at room temperature with agitation with secondary antibodies diluted in 5 % non-fat dry milk in PBS-T 0,1 %: anti-mouse-HRP (1:5000, R&D Systems Cat# HAF007) and anti-rat-HRP (1:2000, Cat# A00167). Membranes were then washed three times, for 10 min each, with PBS-T 0,1 % and incubated with Clarity™ Western Enhanced chemiluminescence (ECL) Substrate (BIO-RAD), according to the manufacturer's recommendations. Finally, the membranes were imaged using the ChemiDoc MP Imaging System (Bio-Rad) and band intensities were quantified using ImageJ software (NIH). The band intensity of uPA was normalized to Alix band intensity for patient samples.

### 2.9 Statistical analysis

Data were expressed as mean ± standard deviation (±SD). The Gaussian distribution of the samples was tested, and the statistical significance of the data was assessed using one-way ANOVA (Tukey post-test) and the unpaired *t*-test was applied to obtain the statistical significance of the means. The P value is indicated in the figures, and statistical significance was considered when p < 0.05. Each experiment was performed in three independent experiments with triplicate. Data were obtained using macrophages from at least 3 different donors.

## 3 Results

### 3.1 TiO_2_ NPs internalize in multivesicular bodies, the exosomes nascent

**Fig. 1:**
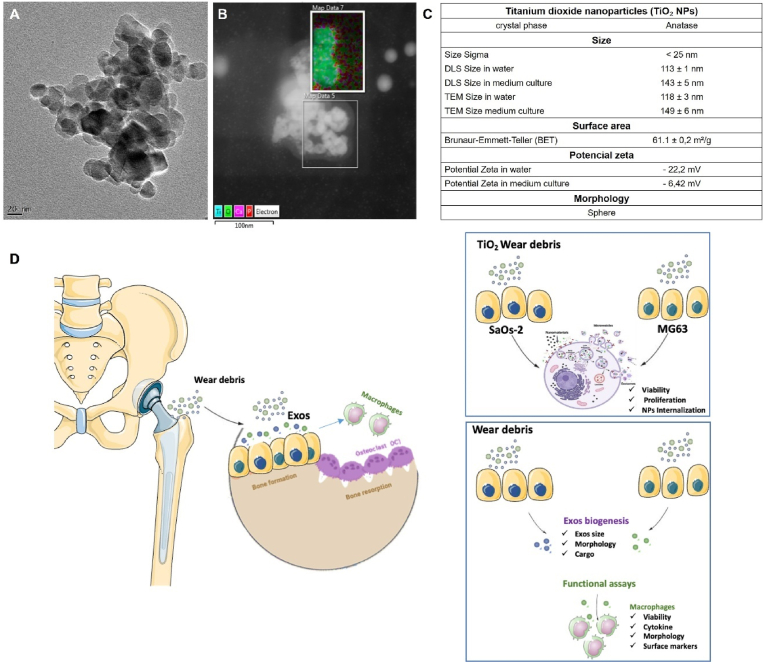
**Characterization of TiO**_**2**_**NPs**: (A) Transmission electron micrograph (TEM) showing the primary size of TiO_2_ NPs and its agglomeration in water after 24 h of dispersion (scale bar: 20 nm). (B) TEM and STEM-EDS element map showing TiO_2_ NPs in medium culture with calcium (Ca) and phosphorus (P) adsorption (scale bar: 100 nm). (C) Table with the main physical and chemical characteristics of the TiO_2_ NPs in water and medium culture. (D) Schematic representation of the workflow (created in Biorender): exposure of TiO_2_ NPs to pre (MG63) and mature osteoblasts (SAOS-2), isolation of Exos and functional tests on macrophages.

Commercially available TiO_2_ NPs mimicking nano wear debris released by titanium prostheses were used in this work. TiO_2_ NPs revealed a round-shaped morphology with a primary size of 25 nm (Fig. 1A). In a culture medium, TiO_2_ NPs agglomerate into structures of about 140 nm with specific proteins and ions adsorbed on them (Fig. 1B), as already reported [21]. The main physicochemical characteristics of TiO_2_ NPs are shown in Fig. 1C. As a proof-of-concept, we investigate the effect of TiO_2_ NPs on osteoblast viability and internalization using two human osteoblastic-like cell lines with different degrees of maturation (Fig. 1D). SAOS-2 and MG63 cell lines representing mature and immature osteoblasts are widely used in bone research [26,27]; they have been used to explore osteoblasts’ reactions to different wear particles [3,4,21] and recently have been employed in fundamental studies of bone cells communication (extracellular vesicles) [16,24,28,29]. A schematic illustration of the experimental rationale is presented in Fig. 1D.

**Fig. 2:**
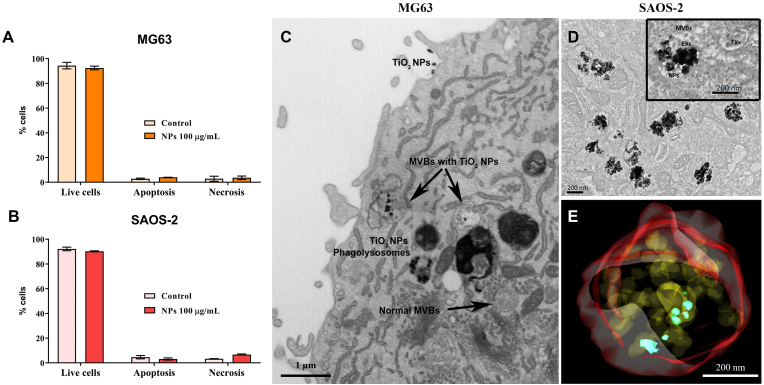
**The effect of TiO**_**2**_**NPs on osteoblasts behavior:** (A) Effect of TiO_2_ NPs on MG63 (immature osteoblasts) and (B) SAOS-2 (mature osteoblasts) cell viability/apoptosis/necrosis (flow cytometry analysis) stimulation. Transmission electron representative micrographs of TiO_2_ NPs internalization on (C) MG63 and (D) SAOS-2 cells revealing internalization of NPs in multivesicular bodies (MVBs). (E) NPs enter in direct contact with Exos as it can be observed ina 3D reconstruction of the vesicles obtained by focus ion beam. (Scale bar: 1 μm, 400 nm, and 200 nm). The results represent the mean ± standard deviation of an independent experimental performed in triplicate.

Our data demonstrated that TiO_2_ NPs did not compromise osteoblast viability (Fig. 2A–B). Interestingly, in both osteoblast models (immature osteoblasts (MG63) and mature osteoblasts (SAOS-2)), TiO_2_ NPs were preferentially located within multivesicular bodies (MVBs), the nascent of Exos (Fig. 2C and D). High-resolution images and 3D reconstruction obtained by focusing ion beam reveal TiO_2_ NPs isolated in multivesicular bodies (MVBs) in direct contact with Exos (Fig. 2E). Considering that TiO_2_ NPs are internalized and share the same vesicle with Exos, we next asked whether TiO_2_NPs could influence osteoblast-derived Exos secretion.

### 3.2 TiO_2_ NPs stimulate osteoblasts-derived exosome secretion and alter their proteomic cargo

**Fig. 3:**
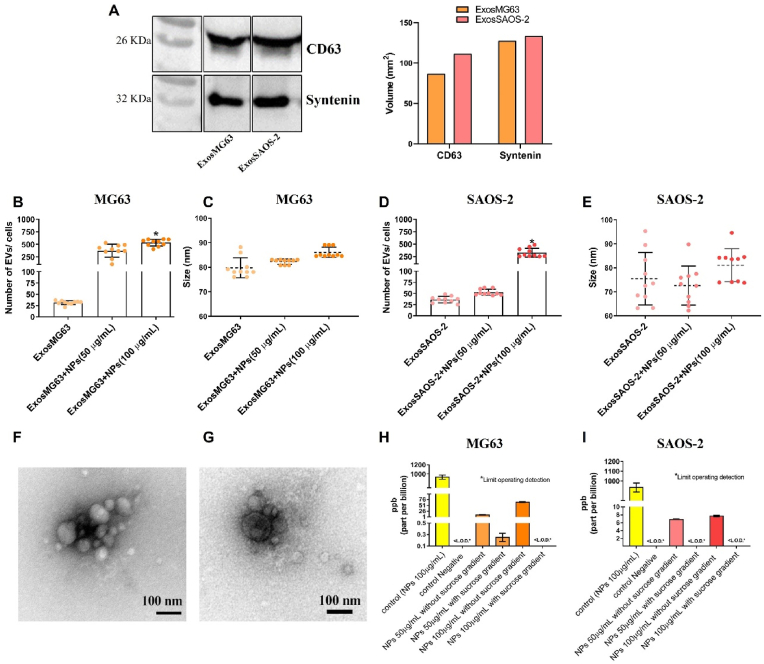
**TiO**_**2**_**NPs stimulated an increase in exosomes secretion:** Exos derived from MG63 (ExosMG63 + NPs) and SAOS-2 (ExosSAOS-2+NPs) after exposure to different concentrations of TiO_2_ NPs, where ultracentrifugation and a sucrose gradient was employed. As a control, the same isolation protocol was used without NP exposure (ExosMG63 and ExosSAOS-2). (A) Identification and quantification of CD63 and Syntenin marker by western blot and densitometry analysis confirming the isolation of Exos derived from MG63 and SAOS-2. (B) Number and (C) size of Exos derived from MG63 and SAOS-2 (D and E) exposed or not (control) to TiO_2_ NPs obtained by nanoparticle size analysis. (F and G) Representative micrographs obtained by Transmission Electron Microscopy revealing the morphology of Exos derived from MG63 and SAOS-2 stimulated with TiO_2_ NPs. Quantification of titanium traces by ICP-MS in Exos derived from (H) MG63 and (I) SAOS-2 (pre-incubated or not with TiO_2_ NPs) after sucrose gradient purification. *ppb (part per billion). Statistical analyzes were performed by ANOVA, followed by multiple comparisons. The results represent the mean ± standard deviation of at least 5 independent experiments in triplicate. *Statistical differences between the groups marked.

Osteoblasts-derived Exos (control without NPs (ExosMG63/ExosSAOS-2) and treatment with TiO_2_ NPs (ExosMG63 + NPs/ExosSAOS-2+NPs)) were isolated using established ultracentrifugation methods [16,24,25] and purified with sucrose gradient to eliminate any trace of TiO_2_ NPs. The isolated Exos were analyzed regarding the expression of Exos-related markers (western blot - WB), size and concentration (TEM and NTA). SAOS-2 and MG63-derived Exos express CD63 and Syntenin marker (Fig. 3A), and according to TEM and NTA analysis, they present a cup-shaped morphology with a diameter ranging from 80 ± 4 nm to 75 ± 5 nm (Fig. 3B–G). Interestingly, Exos secretion increased upon TiO_2_ NPs stimulation in both cell lines, as observed in Fig. 3B and D (see additional files 2A and B), where different concentrations of TiO_2_ NPs exposure were tested. No significant differences were observed in Exos diameter when osteoblastic cells were exposed to TiO_2_ NPs, as evidenced by NTA results (Fig. 3C and E). Transmission electron micrographs reveal (Fig. 3F and G) that TiO_2_ NPs did not alter Exos morphology and that the same nanoparticles could not enter Exos. Inductively coupled plasma spectroscopy analysis of isolated Exos (Fig. 3H and I) confirmed that using a sucrose gradient efficiently obtained Exos-free of titanium and that NPs did not enter Exos.

**Fig. 4:**
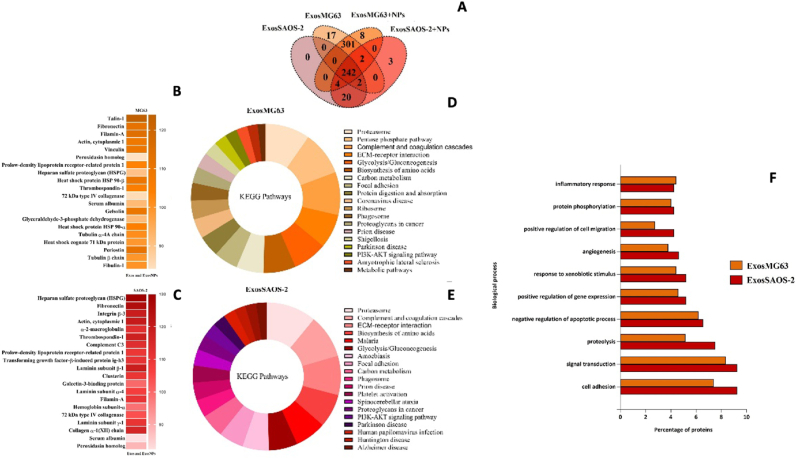
**MG63 and SAOS-2 derived exosomes cargo:** (A) Veen diagram of identified proteins from Exos derived from MG63 and SAOS-2 exposed or not (ExosMG63 and ExosSAOS-2) to TiO_2_ NPs (ExosMG63 + NPs and ExosSAOS-2+NPs) by LC-MS/MS. Top twenty most expressed exosomal proteins (present in both control and treatment) derived from (B) MG63 and (C) SAOS-2 (with different abundance levels (the more intense the color, the greater the abundance of the protein)); (D and E) KEGG pathways classification and (F) biological process of MG63 and SaOs-2 derived Exos. The samples were analyzed in the Uniprot database for taxonomic selection towards *Homo sapiens*. (For interpretation of the references to colour in this figure legend, the reader is referred to the Web version of this article.)

To characterize the potential differences in the protein cargo among the Exos released from both cells' lines, we characterized the isolated Exos by liquid chromatography/tandem mass spectrometry (LC-MS/MS)- based proteomics. In total, 636 proteins for MG63 and 276 for SAOS-2 (Fig. 4A; Figure additional files 3) were identified. The top 20 proteins identified in controls (ExosMG63 and ExosSAOS-2) and TiO_2_ NP treatment (ExosMG63 + NPs and ExosSAOS-2+NPs) are presented in Fig. 4B and C. KEGG pathways of the relevant proteins showed an increase in proteasome, complement and coagulation cascades, pentose phosphate pathway, and ECM-receptor interaction (Fig. 4D and E). Gene ontology cellular components of the most abundant proteins reveal that most were involved in cell adhesion, signal transduction, and proteolysis, among others (Fig. 4F). It is worth mentioning that exosomal proteins from both cell models contained signalling molecules known to play a role in inflammatory response. In addition to these overlapping proteins, Exos of each cell type showed some unique or highly enriched proteins compared to the treatment condition. As observed in the Veen diagram, seventeen proteins were identified exclusively in ExosMG63 (most abundant proteins: filamin-B, thrombospondin-3, matrix Gla protein, septin-7), and eight proteins were solely identified (alpha-N-acetylglucosaminidase, septin-6, tubulin alpha-1C chain, glutathione S-transferase P, among others) after treatment with NPs (ExosMG63 + NPs). In the SAOS-2-derived Exos, three exclusive proteins were found in the control (matrix Gla protein, X-ray repair cross-complementing protein 6, and nucleophosmin), while no proteins were found exclusively in the treatment (ExosSAOS-2+NPs) [16].

**Fig. 5:**
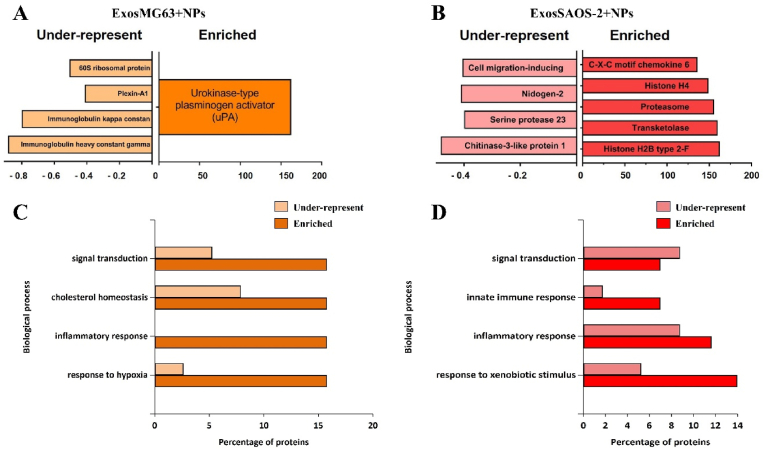
**TiO**_**2**_**nanoparticles alter MG63 and SAOS-2 derived Exos cargo**: Identified clusters that are unique and that are under-represented and/or enriched in Exos derived from (A) MG63 exposed to NPs (ExosMG63 + NPs) and (B) SAOS-2 (ExosSAOS-2+NPs). The biological process was classified by the Uniprot database for taxonomic *Homo sapiens* from (C) MG63 and (D) SAOS-2 cells. Considering the control (p 0.01), a list of proteins that increased and decreased after the treatment of TiO_2_ NPs was identified.

Treatment of osteoblasts with TiO_2_ NPs resulted in the enrichment and under representation of specific exosomal proteins. MG63 derived Exos (Fig. 5A) pre-incubated with TiO_2_ NPs were enriched in urokinase-type plasminogen activator (uPA) whereas 60s ribosomal protein, plexin-A1, immunoglobulin kappa constant and immunoglobulin heavy constant gamma were under-represented. The enriched exosomal proteins in the SAOS-2 model upon NPs exposure were C-X-C motif chemokine 6, Histone H4, Proteasome, Transketolase and Histone H2B type 2-F. On the contrary, cell migration-inducing, nidogen-2, serine protease 23 and chitinase-3-like protein one was under-represented (Fig. 5B). The biological processes of most of the identified enriched proteins were also related to inflammatory responses, innate immune responses, signal transduction, response to xenobiotic stimulus, and response to hypoxia (Fig. 5C and D). As uPA was already reported in literature to be up-regulated in periprosthetic tissues and on macrophages that phagocytosed particles in periprosthetic tissues, functional tests were performed simulating human macrophages with different concentrations of MG63-derived Exos.

### 3.3 Osteoblast-derived exos exposed to TiO_2_ NPs alter the cytokine profile secreted by human macrophages

**Fig. 6:**
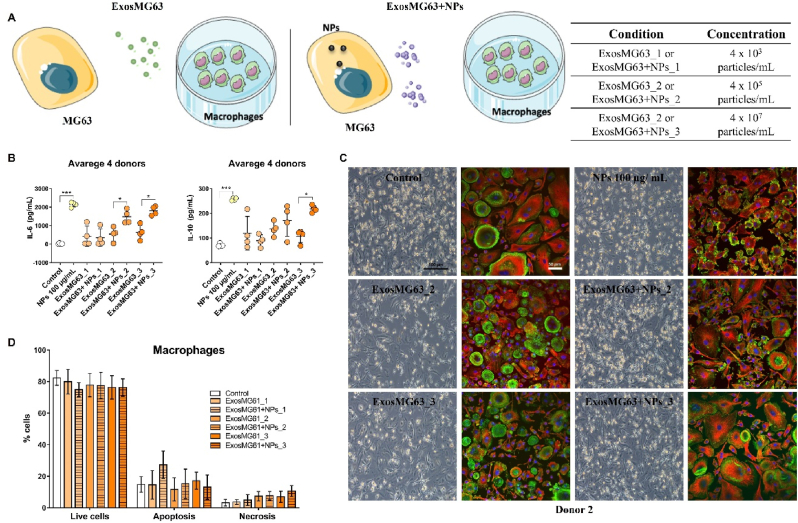
**Exos derived from immature osteoblasts human (MG63) induce the secretion of pro and anti-inflammatory cytokines by macrophages:** (A) Schematic representation of the treatment of macrophages with Exos derived from MG63 after exposure to different concentrations of TiO_2_ NPs (ExosMG63 + NPs_1: 5) and without exposure to NPs (ExosMG63_1:5). Three concentrations of Exos derived from MG63 not exposed (ExosMG63_1:3) and exposed to TiO_2_ NPs (ExosMG63 + NPs_1:3) were used to stimulate human macrophages: 4 x 10³ particles/mL (ExosMG63_1 and ExosMG63 + NPs_1), 4 x 10^5^ particles/mL (ExosMG63_2 and ExosMG63 + NPs_2) and 4 x 10^7^ particles/mL (ExosMG63_3 and ExosMG63 + NPs_3). As control of all the experiments, we analyzed macrophages not exposed (control) and exposed to 100 μg/mL of TiO_2_ NPs during 72 h (NPs 100 μg/mL). (B) Human macrophages from 4 healthy donors were exposed to different concentrations of MG63 derived Exos upon which pro- (IL-6) and anti-inflammatory (IL-10) cytokines were determined by ELISA in the supernatants. Data are presented as averages obtained from the 4 donors. (C) Representative phase-contrast (scale bar: 100 μm) and confocal microscopy images (scale bar: 50 μm) of macrophages stimulated or not (control) with Exos derived from MG63. The macrophage's actin cytoskeleton is illustrated in green, tubulin in red, while nuclei are counterstained with DAPI (in blue). (D) Macrophage viability upon MG63-derived Exos treatment was determined by flow cytometry. Statistical analyses were performed by ANOVA, followed by multiple comparisons, using the GraphPad Prism software. The results represent the mean ± standard deviation of at least three independent experiments performed in triplicate. *Statistical differences between the groups marked. (For interpretation of the references to colour in this figure legend, the reader is referred to the Web version of this article.)

To assess the influence of TiO_2_ NPs on bone-immune cells crosstalk, functional assays were performed exposing different amounts of Exos derived from MG63 bone cells that were stimulated (ExosMG63 + NPs_1:3) or not (ExosMG63_1:3) with 100 μg/mL TiO_2_ NPs to primary human macrophages (see the scheme presented in Fig. 6A). Pro-inflammatory (interleukin 6 (IL-6), tumor necrosis factor α (TNF-α), interleukin 1 beta (IL-1 β)) and anti-inflammatory (interleukin 10 (IL-10), transforming growth factor-β (TGF-β)) cytokines, macrophage viability, cytoskeleton organization and polarization surface markers were evaluated. The higher concentration of osteoblast-derived Exos (ExosMG63 + NPs_3) induced a significant increase in human macrophage secretion of both IL-6 and IL-10 (Fig. 6B and Figure in additional files 4A). Individual cytokine results presented by donors are shown in Figure in additional files 4. These results indicate that Exos-derived from osteoblast cells treated with TiO_2_ NPs specifically induced macrophage activation, unlike their counterparts (ExosMG63_1:3). Detailed microscopic analysis revealed alterations in macrophage morphology (Fig. 6C and Figure in additional files 5), a known indicator of macrophage phenotype/activation status [30], as evidenced by the alterations between TiO_2_ NPs-treated and untreated macrophages (control). Despite this, Exos did not alter the viability of macrophages (Fig. 6D). Comparing the effect of Exos with the direct contact of TiO_2_ NPs on macrophage behavior, it was possible to observe similar results except for macrophage viability that was compromised due to ROS overproduction (Figure in additional files. 6A and B) and increased lysosomal activity (Figure in additional files. 6C) triggered by the internalization of NPs (Figure in additional files. 6D). Resuming the osteoblast derived exosomes stimulated by nanowear debris directs macrophages towards pro-inflammation pathways due to exosome cargo.

**Fig. 7:**
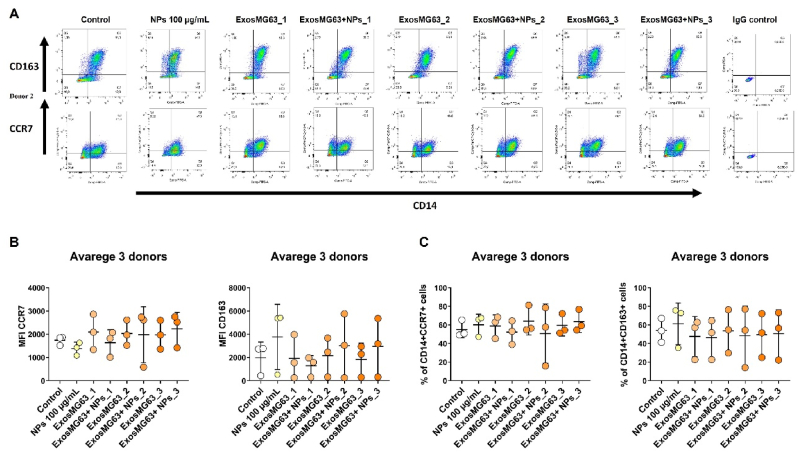
**Osteoblasts-derived Exos induce a mixed macrophage polarization:** Macrophages were differentiated for 10 days and then treated for 72 h with Exos isolated from MG63 cells previously incubated or not with TiO_2_ NPs. By flow cytometry, CD14 was used as a macrophage lineage marker and CD163 and CCR7 were used as M2 and M1-associated markers, respectively. Representative dot plots illustrate the gates used to identify CD14^+^ CD163^+^ cells and CD14^+^ CCR7^+^ cells (FlowJo software). (A) Numbers indicate the percentage of the double-positive populations. (B) The mean fluorescence intensity (MFI) of CCR7 and CD163 was determined by subtraction of the fluorescence intensity of the respective isotype control. (C) The percentage of double-positive cells (CD14^+^CCR7^+^ and CD14^+^CD163^+^) was also determined. Statistical analyzes were performed by ANOVA, followed by multiple comparisons, using GraphPad software. The results represent the mean ± standard deviation of values obtained with macrophages from at least 3 different donors.

In parallel, Exos-derived from osteoblasts pre-incubated with TiO_2_ NPs induced an overall increase in cell area and a less intense cortical actin ring. Specially in the intermediate Exos concentration, a shift in morphology was clearly observed, with a higher density of fusiform macrophages, typical of an M1-like phenotype, as previously described [29]. Our data clearly demonstrates that the observed activation of macrophages is exclusive of Exos and not a dual effect of Exos and NPs. No significant differences were observed in IL-1 β, TNF-α or TGF-β secretion levels (see Figure in additional files 4B) neither on the expression of macrophage phenotype-associated surface markers (Fig. 7A and B, C and Figure in additional files 7A and B), which could be most likely explained by the existence of a mixed M1-M2 macrophage phenotype (as it happens at the implant site) and also to the inherent variability between blood donors.

### 3.4 uPA as a patient-derived exosome candidate biomarker of osteolysis

**Fig. 8:**
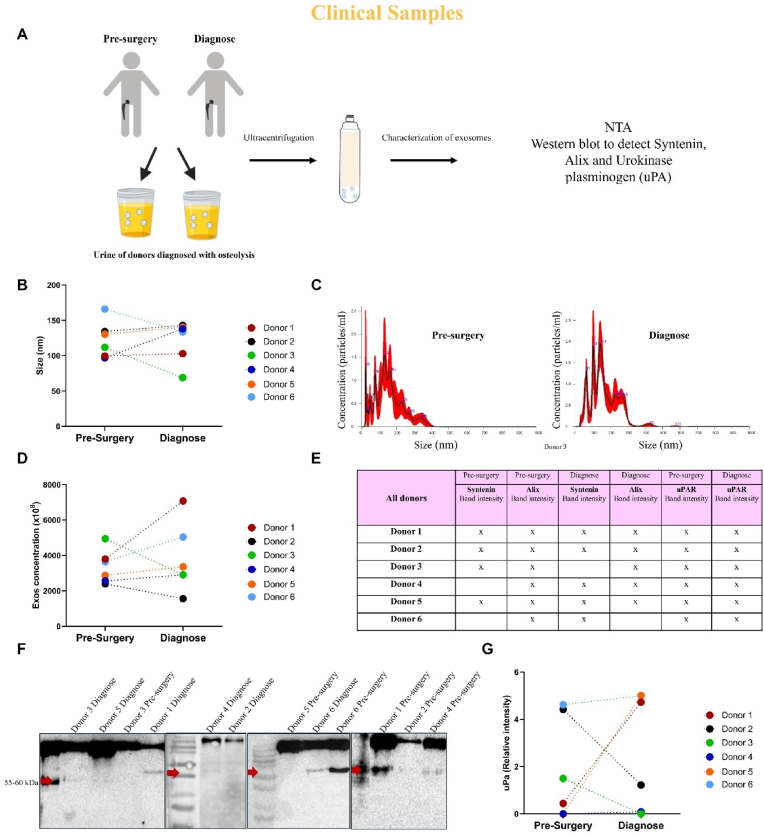
**Characterization of Exos derived from patients with peri-implant osteolysis:** Schematic illustration of the experimental set-up for obtaining a clinical sample and the characterization of the exosomes obtained (A). Nanoparticle tracking analysis of the Exos population isolated from the urine of patients that undergone hip surgery (pre-surgery) and patients with osteolysis (diagnosis): average size and size distribution (B, and C). Exos concentration in the different time points (D). Table demonstrating the characterization that was performed in Exos regarding specific markers: Alix, Syntonin and uPa (E). Identification and quantification of uPA western blot in Exos from osteolysis patients (F and G).

Next, we isolated Exos from the urine of patients (Donors) before the first hip-replacement surgery (Pre-surgery), and when osteolysis was radiographically documented (Diagnosis) (Fig. 8A). NTA analysis showed extracellular vesicles of 134,5 ± 26 nm (mean ± s.d.) (Fig. 8B–and C). No significant differences were observed in the size nor in the concentration of isolated exosomes at the different time points (Fig. 8C and D). In addition, Exos are positive for exosome markers such as Alix (Figure in additional files. 8) and syntenin-1 (Figure in additional files. 8). uPA was expressed in Exos in the urine of osteolytic patients at both time points, before surgery and at the time of diagnosis (Fig. 8E and F). However, there was no difference in the relative intensity between the two-time points (Fig. 8G).

**Fig. 9:**
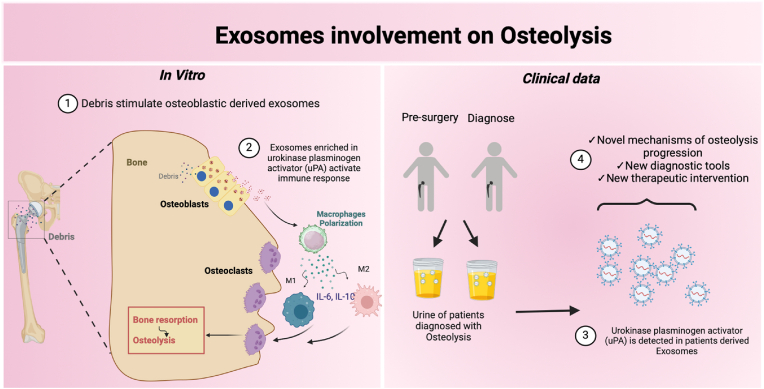
**Schematic representation demonstrating the involvement of exosomes in osteolysis:** Titanium implantable systems, due to wear processes in the human body start to release nanowear debris at the interface implant bone. These TiO_2_ NPs interact with osteoblasts with different degrees of maturation at the bone-implant interface. These TiO_2_ NPs are internalized by bone cells and localized directly in MVBs, altering Exos biogenesis and cargo (1). Immature osteoblasts derived Exos (from cells previously stimulated to TiO_2_ NPs) enriched with uPA stimulate macrophages towards an inflammatory pathway already reported in peri-implant osteolysis (2). The isolation of Exos from the urine of patients with osteolysis reveals the expression of uPA (3) opening venues for the possible employment of uPA as a candidate biomarker of the disease (4). Scheme created in Biorender.

## 4 Discussion

The wear of modular junctions releases a wide range of debris with different chemical compositions, sizes, and shapes [3,4,16,28,31]. From the literature, titanium wear particles usually have a round shape with a size ranging from 51 to 116 nm, and the anatase crystal phase is the most abundant crystal phase in periprosthetic tissues, especially in bone marrow [4,32]. We have previously reported that TiO_2_ NPs (anatase crystal structure, primary size <25 nm), similar in size to the wear particles associated with prosthetic implants, were internalized by primary human osteoblasts, and triggered the release of proinflammatory cytokines that contribute to the inflammatory processes involved in particle-induced osteolysis [3,4,8,19]. However, at the implant-bone interface, several differentiation processes occur during bone healing in which wear debris comes into direct contact with mature and immature osteoblasts and other cells [2,[20], [21], [22]]. To understand how osteoblasts with different maturation levels behave in the face of exposure to TiO_2_ NPs, MG63 and SAOS-2 cell lines were used and stimulated with 100 μg/mL TiO_2_ NPs during 72 h. The results show that both cell lines respond similarly to exposure to TiO_2_ NPs. A detailed description of the cellular and molecular mechanisms underlying the biological response to titanium implant wear particles has been extensively described in the literature [1,4,9,11,31]. Interestingly, TiO_2_ NPs in both cell lines preferentially internalize into multivesicular bodies, the nascent of Exos. This finding suggests that maturation of TiO_2_ NPs in endosomes can occur by two independent pathways: i) fusion of MVBs containing TiO_2_ NPs with lysosomes, where acidic compartments provide degradation of their contents (although TiO_2_ NPs are not degraded due to their physicochemical properties); ii) MVBs can also fuse with the plasma membrane and release their contents as Exos into the extracellular space [[13], [14], [15],^33^,^34^]. Details of the basic mechanisms of MVB and Exos generation have been elucidated in several publications. However, the mechanisms underlying the biogenesis of EVs upon exposure to TiO_2_ NPs and the sorting of these molecules remain to be explored [25,[33], [34], [35]].

Accumulating evidence has revealed the role of osteoblast-derived Exos in mediating intercellular crosstalk in bone remodelling. They facilitate a diversity of intracellular and intercellular signalling cascades that regulate osteoclastogenesis and osteogenesis [22,33]. Our previous study demonstrated that osteoblast-derived Exos decreased osteogenic differentiation of mesenchymal stem cells upon internalization of TiO_2_ NPs [16]. This suggests that osteoblast-derived Exos play a vital role at the bone-implant interface and enhances the adverse effects of wear particles, which may lead to periprosthetic bone loss. However, the possible contribution of Exos cargo to the systemic diffusion of toxic signals and activation of the innate immune system remains to be elucidated [8,25].

Exos are essential mediators of cell-cell communication, and their biogenesis and fate can be altered by physical and chemical factors [14]. The application of Exos research in toxicology is still recent, with few studies showing that Exos provides a mechanistic link between inhalation exposure, airway inflammation, and systemic effects [14,36]. Our results demonstrate the successful isolation of Exos from different osteoblast populations and that TiO_2_ NPs induce a dose-dependent secretion of Exos, whereas TiO_2_ NPs do not internalize Exos. These results are consistent with the literature, which has previously reported that Exos secretion, intravesical cargo, and activity are stimulated by exogenous physical and chemical factors, including stimulation by nanomaterials, oxidative stress, pH variations, and hypoxia, among others [37]. It is important to keep in mind that the internalization and encapsulation of NPs in exosomes are determined by the primary size of the NPs and their agglomeration in the cell culture medium. Other studies have already shown that NPs (Fe_3_O_4_ NPs, MIONs, Ag–Fe NPs, among others) are not internalized into exosomes due to the size limitations of the intraluminal vesicles of multivesicular bodies. In this article, TiO_2_ NPs have an average size of 145 ± 5 nm, and the average size of exosomes was about 80 ± 4 nm, it was not expected that TiO_2_ NPs could enter exosomes.

The common proteins present in Exos from both conditions were involved in cell adhesion, signal transduction, proteolysis, and signalling molecules known to play a role in inflammatory response. Besides that, Exos secreted by immature and mature osteoblasts were enriched in cytoskeletal proteins, among the most abundant components of extracellular vesicles. Since Exos fuse with target cells, it is possible that the presence of cytoskeletal elements on Exos could enhance cellular uptake and cellular responses, such as direct cell movement [16]. The involved mechanisms in sorting proteins in Exos (ESCRT, tetraspanins, and lipid-dependent mechanisms) have already been explored in literature [16,18,38].

The gradual inflammatory response to wear particles of titanium implants is the hallmark of periprosthetic osteolysis [16,17,31,33]. Innate immune implant debris-induced inflammation is caused predominantly by macrophages that, together with inflammatory micro-environmental signals, induce an M1‐like macrophage polarization, exacerbating the production of inflammatory signals (IL-8, monocyte chemotactic protein-1 (MCP- 1), and MIP-1, interleukin (IL)-6, IL-1β, tumor necrosis factor α (TNF-α) that favors osteoclastogenesis with consequent prothesis failure [9,[39], [40], [41], [42]]. Not only M1 macrophages release inflammatory mediators and catabolic enzymes that drive the inflammatory process, but M2 macrophages also produce TGF-β, IL10, and other signalling molecules to resolve inflammation and initiate tissue regeneration [41,[43], [44], [45], [46]]. Clinical studies show that end-stage osteolysis is characterized by an unconventional macrophage activation pathway in periprosthetic tissues, characterized by generating a specific cytokine and chemokine milieu but not by increased levels of proinflammatory cytokines [46]. The pro-inflammatory responses may be transient, with M2 responses displaced in the later stages of disease progression [[44], [45], [46]].

Interestingly, we observed that the higher concentration of MG63-derived Exos stimulated to NPs (ExosMG63 + NPs) triggered a significant increase in the secretion of both IL-6 and IL-10 from human macrophages, indicating an M1-M2 mixed phenotype already reported in osteolysis (see Fig. 9). The precise mechanisms for M1-M2 mixed phenotype upon exposure to MG63-derived Exos are still unknown; however, one possibility is the Exos cargo that has a unique content when stimulated with TiO_2_ NPs. MG63-derived Exos contains eight proteins exclusive to the treatment (ExosMG63 + NPs), with three involved in inflammatory processes. Septin-6 is involved in macrophage shape and polarity regulation, receptor signalling, cytokinesis, cell migration, and vesicle transport [47]. In addition, glutathione S-transferase P, a cytosolic, mitochondrial, and microsomal enzyme that prevents cellular damage from deleterious stimuli of xenobiotic metabolites, has been previously described as an activator of the macrophage-mediated inflammatory response [48]. Fascin functions regulate cytoskeletal structures for maintaining cell adhesion, coordinate motility and cell invasion, and play an essential role in immunosuppression by triggering a mixed inflammatory response mediated by macrophages [49]. Besides that, ExosMG63 + NPs were enriched in urokinase plasminogen activator, an enzyme that catalyzes the conversion of plasminogen to plasmin. Immunohistochemistry results have previously reported in the literature the localization of uPA in macrophages of periprosthetic tissue that phagocytosed metal, polyethylene, and cement particles or accompanying necrotic bone pieces [50,51]. These results suggest that non-degradable debris may trigger a proteolytic activation cascade that may contribute to prosthesis loosening [52]. Moreover, uPA expression before hip replacement surgery and at diagnostic time confirms that the uPA/uPAR system is active in osteolysis as well as other inflammatory diseases, including osteoarthritis (the primary cause of hip replacement), and may serve as a modulator of immunological responses [51,[53], [54], [55], [56]].

Interestingly, upregulation of uPA has already been found in the pseudocapsular tissue and interface tissue around implants in patients with total hip prosthesis loosening [57,58] and in osteoblast cultures derived from patients with osteoarthritis and in osteoblast cultures derived from patients after failed joint replacement surgery [51]. Resuming, the fact that Exos derived from peri-implant osteolysis patients express uPA, suggests that uPA can be a possible candidate circulating biomarker for the early detection of osteolysis. However, the clinical validation of uPA was conducted on a limited number of patient samples. Increasing the sample size and including a more diverse patient population would enhance the robustness of the results. Future work is necessary to test this biomarker in independent and multicentric cohorts and to determine their clinical utility to predict osteolysis before substantial peri-implant bone loss. Longitudinal studies prospectively collected from patients following total joint replacement surgery and tracking biomarker changes over time in patients with joint implants could provide deeper insights into the progression of peri-implant osteolysis and the predictive value of identified biomarkers. Finally, the study did not extensively account for potential confounding factors such as patient comorbidities, medication use, and lifestyle factors that could influence exosome content and biomarker levels.

Resuming, our data suggest that Exos derived from immature osteoblasts exposed to TiO_2_ NPs modulate macrophages toward a mixed inflammatory profile reported in particle-induced osteolysis [3]. These results are clinically relevant because they demonstrate for the first time that TiO_2_ NPs, with their low solubility, can accumulate in the multivesicular bodies of osteoblast cells and affect Exos biogenesis. Moreover, direct contact with TiO_2_ NPs is not required to stimulate macrophage-dominated inflammatory responses, as Exos with their specific cargo elicits a mixed inflammatory profile. Our results suggest that monocytes recruited to the implant site by inflammation or resident macrophages in the surrounding tissue may be activated by osteoblasts-derived Exos that have been in contact with nanowear debris.

There are still too many unanswered questions. To date, we have yet to fully understand the effects of the physicochemical properties of wear debris on Exos biogenesis and cell communication. However, we would like to emphasize that the findings presented in this article highlight a previously unrecognized mechanism of communication network established in the periprosthetic niche that leads to osteolysis establishment. That goes beyond the proposal of a novel mechanism of osteolysis progression and opens an unprecedented possibility of identifying novel disease biomarkers (exosome cargo including proteins but also microRNA). A comprehensive characterization of the microRNA landscape and its functional implications is necessary to strengthen our findings, providing a more holistic understanding of the molecular mechanisms underlying wear particle-induced osteolysis.

The foreseen scientific and technological discoveries of this article can contribute to revolutionize the early detection of osteolysis by developing the next generation of point-of-care devices for osteolysis, which will give healthcare professionals continuum monitoring of patients and will drastically define new personalized joint arthroplasty disease management. Furthermore, osteolysis is considered a representative condition of other osteolytic lesions, such as osteoporosis, osteosarcoma, and osteoarthritis, bringing new knowledge to those pathologies and enlarging the impact on the clinical onset. Besides that, exosomes can be tailored to deliver specific cargo targeted to improve joint repair and regeneration, contributing to improving the therapy success rate and patient recovery. This goes beyond traditional symptomatic relief, minimizing side effects and leading to more sustainable and long-lasting treatment. The modular nature of engineered exosomes (e.g., genetic engineering approaches) opens opportunities for combinatorial therapies (Exosomes designed to carry multiple therapeutic agents to address different aspects of the disease) as well as reduce antibiotic resistance concerns.

## 5 Conclusions

This study unravels the distinctive influence of TiO_2_ NPs, mimicking nanoscale wear debris, on osteoblast exosome secretion and their interplay with immune cells. TiO_2_ NPs enter multivesicular bodies, the nascent of Exos of both bone cell lines altering Exos biogenesis. The size, shape, and morphology of Exos were similar; however, a distinct exosomal (enriched in uPA) cargo was observed upon TiO_2_ NPs stimulus. A higher concentration of osteoblast-derived Exos enriched in uPA-induced macrophages towards a mixed inflammatory profile that has been reported in particle-induced osteolysis. Urinary exosomes derived from osteolysis patients reveal the expression of uPA suggesting that it may have the potential as a biomarker of osteolysis.

## Ethical approval and consent to participate

Human cell samples: All studies involving human cell samples were approved by the CHUSJ Ethics Committee for Health (References 259 and 260/11) in accordance with the Helsinki Declaration. Informed consent was obtained from all participants. Human urine samples: The Institutional Review Board for Human Research approved the study design. Each patient provided consent for their biofluids to be stored for future research projects. All studies using these samples were approved by the Rush Medical Center Ethics Committee (Reference 14102805), in compliance with the Helsinki Declaration.

## Consent for publication

Not applicable.

## Availability of data and materials

The datasets used and/or analyzed during this study are available from the corresponding authors on reasonable request.

## Funding

This research was funded by the European Union's H2020 project Sinfonia (no. 857253). A. R. Ribeiro especially thanks to Propesq-Unigranrio-FUNADEP Scholarship and Jovem Cientista do Nosso Estado award from 10.13039/501100004586FAPERJ (E−26/203.179/2017 (232897). J M G thanks Cientista do Nosso Estado award from 10.13039/501100004586FAPERJ. 10.13039/100021243SAM laboratory is supported by European Regional Development Fund (10.13039/501100008530ERDF) through COMPETE 2020 – Operacional Programme for Competitiveness and Internationalization (POCI), Portugal 2020, and by FCT – Fundacao para a Ciencia e a Tecnologia (FCT)/Ministério da Ciencia, Tecnologia e Inovacao in the framework of the projects POCI-01-0145-FEDER-032189 and UIDB/50006/2020. W Souza states that this study was funded by 10.13039/501100004586FAPERJ - Fundacao Carlos Chagas de Amparo à Pesquisa do Estado do Rio de Janeiro, Process Sei E−26/204.586/2021 and 204.587/2021 (268814), and by Capes/FTC – 88887.163123/2018–00, for which we are particularly grateful to these funders. L.A. Rocha acknowledges the financial support from projects CAPES 99999.008666/2014-08, FAPESP M.ERA-NET, Proc. 2015/50.280-5 and FAPESP Proc. 2017/24300-4.

## CRediT authorship contribution statement

**Wanderson de Souza:** Writing – review & editing, Writing – original draft, Visualization, Validation, Resources, Methodology, Investigation, Formal analysis, Data curation, Conceptualization. **S. Gemini-Piperni:** Writing – review & editing, Writing – original draft, Methodology, Investigation, Formal analysis, Data curation. **Carolina Ruivo:** Methodology, Investigation. **Nuno Bastos:** Methodology, Investigation. **Sofia Almeida:** Methodology, Investigation, Data curation. **Daniel Lopes:** Validation, Methodology, Formal analysis. **Patricia Cardoso:** Writing – original draft, Validation, Methodology, Investigation. **Maria Jose Oliveira:** Supervision, Funding acquisition, Formal analysis. **D. Rick Sumner:** Writing – review & editing, Validation, Supervision, Investigation. **Ryan D. Ross:** Writing – review & editing, Supervision, Data curation. **Joshua J. Jacobs:** Data curation, Formal analysis, Investigation, Methodology. **Jose Mauro Granjeiro:** Writing – original draft, Visualization, Validation, Supervision, Software, Resources, Methodology, Investigation, Funding acquisition, Formal analysis, Data curation, Conceptualization. **Maria Helena Fernandes:** Writing – review & editing, Writing – original draft, Supervision, Investigation, Funding acquisition, Formal analysis. **Luis A. Rocha:** Validation, Supervision, Funding acquisition, Formal analysis, Data curation. **Sonia Melo:** Writing – review & editing, Writing – original draft, Validation, Supervision, Software, Resources, Methodology, Investigation, Funding acquisition, Formal analysis, Data curation. **Ana R. Ribeiro:** Writing – review & editing, Writing – original draft, Visualization, Validation, Supervision, Software, Resources, Project administration, Methodology, Investigation, Funding acquisition, Formal analysis, Data curation, Conceptualization.

## Declaration of competing interest

The authors declare that they have no known competing financial interests or personal relationships that could have appeared to influence the work reported in this paper.

## Data Availability

Data will be made available on request.

## Abbreviations

TiO_2_ NPs: Titanium dioxide Nanoparticles
Exos: Exosomes
uPA: urokinase-type plasminogen activator
MVBs: Multivesicular Bodies
MG63: Human osteosarcoma cell lines
SAOS-2: Human osteosarcoma cell lines
IL-6: Interleukin 6
IL-8: Interleukin 8
TNF-a: Tumor necrosis factor α
IL-1b: Interleukin 1 beta
IL-10: Interleukin 10
TGF-b: Transforming growth factor beta
MCP-1: Monocyte chemotactic protein-1
MIP-1: Macrophage inflammatory protein 1
FN: Fibronectin
TLRs: Toll-like receptor
LBP: Lipopolysaccharide-binding protein
MRP-14: Myeloid-related protein14
ROS: Reactive Oxygen Species
TEM: Transmission Electron Microscopy
NTA: Nanoparticle Tracking Analysis
